# Synergistic Regulation of Ag Nanoparticles and Reduced Graphene Oxide in Boosting TiO_2_ Microspheres Photocatalysis for Wastewater Treatment

**DOI:** 10.3390/nano15191510

**Published:** 2025-10-02

**Authors:** Guoshuai Ma, Zhijian An, Yinqi Yang, Wei Wang, Yao Wang, Shuting Tian, Jingwen Gao, Xue-Zhong Gong, Laurence A. Belfoire, Jianguo Tang

**Affiliations:** 1 Institute of Hybrid Materials, National Center of International Research for Hybrid Materials Technology, National Base of International Science & Technology Cooperation, College of Materials Science and Engineering, Qingdao University, Qingdao 266071, China; 18663687028@163.com (G.M.); anzhijian2024@163.com (Z.A.); 2304825916@qq.com (Y.Y.); wangyao72@qdu.edu.cn (Y.W.); tianshuting1104@163.com (S.T.); 2621757808@qq.com (J.G.); xzgong@qdu.edu.cn (X.-Z.G.); belfiore@engr.colostate.edu (L.A.B.); 2 Department of Chemical and Biological Engineering, Colorado State University, Fort Collins, CO 80523, USA

**Keywords:** TiO_2_ microsphere, photocatalytic degradation, semiconductors, nanocomposites

## Abstract

Dye-contaminated wastewater has become one of the most severe environmental challenges due to the non-biodegradability and toxicity of synthetic dyes. While photocatalytic degradation is considered a green and efficient technology for wastewater purification, conventional TiO_2_ suffers from limited light utilization and rapid electron–hole recombination. In this exploration, Ag-TiO_2_-RGO nanocomposites were successfully fabricated and systematically investigated by XRD, SEM, TEM, XPS, Raman, and PL spectroscopy. The incorporation of Ag nanoparticles and reduced graphene oxide (RGO) synergistically improved charge separation and transfer efficiency. Photocatalytic activity was evaluated using different dyes as pollutants under visible light irradiation. Among the samples, Ag-TiO_2_-RGO-3% exhibited the highest RhB degradation efficiency of 99.5% within 75 min, with a rate constant (K) of 0.05420 min^−1^, which was nearly three times higher than that of pure TiO_2_. The photocatalyst also showed excellent reusability with only minor efficiency loss after five cycles, and its activity remained stable across a wide pH range. Radical trapping experiments revealed that •O_2_^−^ served as the dominant reactive species, with additional contributions from •OH and photogenerated holes (h^+^). A possible photocatalytic mechanism was proposed, in which Ag nanoparticles and RGO effectively suppressed electron–hole recombination and accelerated the formation of reactive oxygen species for efficient dye mineralization. These findings demonstrate that Ag-TiO_2_-RGO-3% is a promising photocatalyst with high activity, stability, and environmental adaptability for wastewater remediation.

## 1. Introduction

With the rapid development of the textile, printing, leather, and paper industries, large quantities of synthetic dyes are discharged into aquatic environments each year [1,2,3]. These dyes typically possess complex aromatic structures, high chemical stability, and strong resistance to natural degradation, which not only lead to persistent water contamination but also pose serious threats to ecosystems and human health [4,5,6]. Conventional wastewater treatment approaches, including adsorption, coagulation, and biological degradation, often fail to completely eliminate these pollutants due to their limited efficiency and the risk of secondary pollution [7,8,9]. Consequently, the development of green, cost-effective, and sustainable strategies for the efficient removal of dye contaminants from water has become an urgent priority [10,11,12].

Among various treatment technologies, semiconductor-based photocatalysts have attracted significant attention as an effective and environmentally friendly method for the degradation of organic dyes [13,14,15,16,17]. This technique utilizes solar or artificial light irradiation to generate electron–hole pairs in semiconductor materials, which subsequently react with water and oxygen to produce reactive oxygen species (ROS), such as hydroxyl radicals (•OH) and superoxide radicals (•O_2_^−^) [18,19,20]. These ROS are highly oxidative and can mineralize organic pollutants into harmless end-products such as CO_2_ and H_2_O. In this context, titanium dioxide (TiO_2_) has emerged as one of the most widely studied photocatalysts due to its low cost, strong oxidation capability, chemical stability, and non-toxicity [21,22,23].

Nevertheless, pristine TiO_2_ still suffers from several limitations that restrict its practical applications [24,25,26]. Most notably, it possesses a wide band gap (~3.2 eV), which restricts its light absorption mainly to the UV region, accounting for less than 5% of the solar spectrum [27,28]. Moreover, the fast recombination rate of photogenerated electron–hole pairs significantly lowers its photocatalytic efficiency. To address these challenges, various modification strategies have been proposed, among which noble metal loading (e.g., Ag, Au, Pt) has shown promising results [29,30,31]. Noble metal nanoparticles not only act as electron sinks, facilitating charge separation and suppressing recombination, but also exhibit localized surface plasmon resonance (LSPR) effects, which extend the light absorption of TiO_2_ into the visible region. Specifically, silver (Ag) nanoparticles are particularly attractive due to their excellent conductivity, moderate cost, and strong plasmonic effects, making them an efficient cocatalyst to enhance TiO_2_ photocatalytic activity [32,33].

Despite these advantages, noble metal-modified TiO_2_ still faces shortcomings, such as aggregation of nanoparticles during the reaction, limited surface area for dye adsorption, and insufficient electron transport efficiency [34,35]. To further optimize the photocatalytic system, the incorporation of two-dimensional (2D) materials has been investigated. Reduced graphene oxide (RGO), as a typical 2D carbon material, offers several unique advantages [36,37]. It provides a large specific surface area and abundant functional groups, which improve the dispersion of TiO_2_ nanoparticles and facilitate dye adsorption. More importantly, RGO possesses excellent electrical conductivity, which enables it to act as an electron acceptor and transporter, thereby accelerating charge separation and prolonging the lifetime of photogenerated carriers [38,39]. Compared with other carbon-based modifiers, RGO also maintains good chemical stability, high transparency, and tunable surface chemistry, making it a promising cocatalyst in photocatalytic applications [40,41].

In this work, an Ag-TiO_2_-RGO ternary nanocomposite was rationally designed and synthesized via a facile strategy. The synergistic interactions among TiO_2_, Ag nanoparticles, and RGO are expected to overcome the intrinsic limitations of pristine TiO_2_. In particular, Ag nanoparticles enhance visible-light absorption and act as electron traps, while RGO sheets provide conductive networks that promote charge transport and suppress electron–hole recombination. As a result, the optimized composite is anticipated to demonstrate superior photocatalytic activity for dye degradation compared with pristine TiO_2_ and binary Ag-TiO_2_ systems. Beyond practical performance, this study also offers fundamental insights into the roles of noble metal nanoparticles and two-dimensional carbon materials in advancing semiconductor photocatalysis, thereby providing a promising strategy for the development of efficient and durable photocatalysts for wastewater treatment.

## 2. Experimental

### 2.1. Preparation of TiO_2_ and Ag-TiO_2_-RGO

**Figure 1:**
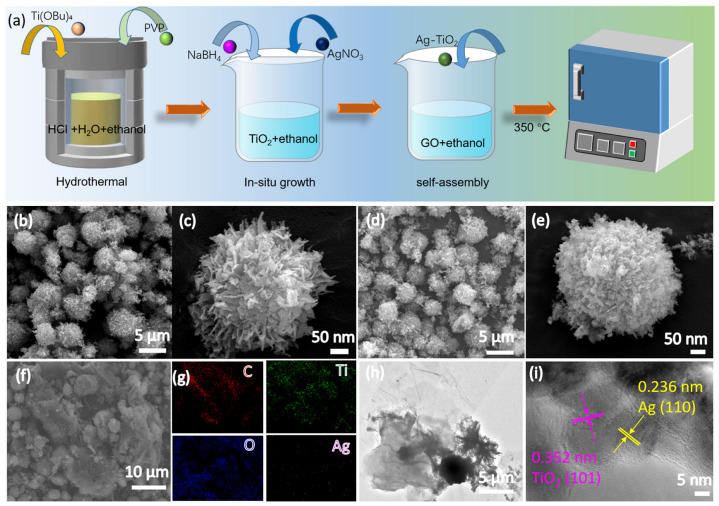
(**a**) Schematic of preparation of Ag-TiO_2_-RGO; SEM images of (**b**,**c**) pure TiO_2_, (**d**,**e**) Ag-TiO_2_, and (**f**) Ag-TiO_2_-RGO-3% and (**g**) corresponding elemental mapping; (**h**) TEM and (**i**) HRTEM of Ag-TiO_2_-RGO-3%.

The preparation process of Ag-TiO_2_-RGO is shown in Figure 1a. Firstly, titanium butoxide [Ti(OBu)_4_] was added dropwise to a mixed solution of ethanol, deionized water, and HCl under vigorous stirring. Polyvinylpyrrolidone (PVP) was introduced as a stabilizer. The resulting mixture was transferred to a Teflon-lined autoclave and heated at 180 °C for 12 h to obtain TiO_2_ nanoparticles. Next, Ag was loaded onto TiO_2_ by in situ reduction. Specifically, AgNO_3_ and NaBH_4_ were sequentially added into the TiO_2_-ethanol dispersion under constant stirring, leading to the formation of Ag-TiO_2_. Subsequently, Ag-TiO_2_ was mixed with a graphene oxide (GO) suspension in ethanol and sonicated to achieve uniform self-assembly. The obtained Ag-TiO_2_-GO composite was dried and then annealed at 350 °C in air for 2 h to reduce GO to RGO, yielding the final Ag-TiO_2_-RGO product. To investigate the effect of different RGO content on photocatalytic degradation performance for Ag-TiO_2_-RGO, various weight ratios of RGO in Ag-TiO_2_-RGO were fabricated, including Ag-TiO_2_-1 wt% RGO, Ag-TiO_2_-2 wt% RGO, Ag-TiO_2_-3 wt% RGO and Ag-TiO_2_-4 wt% RGO, termed as Ag-TiO_2_-RGO-1%, Ag-TiO_2_-RGO-2%, Ag-TiO_2_-RGO-3% and Ag-TiO_2_-RGO-4%, respectively.

### 2.2. Characterization

The morphology and internal structure of the prepared materials were analyzed using scanning electron microscopy (SEM, Hitachi S-4800, Hitachi High-Technologies, Tokyo, Japan) and transmission electron microscopy (TEM, JEOL JEM-2100, JEOL Ltd., Tokyo, Japan) equipped with selected area electron diffraction (SAED). The phase composition was identified by X-ray diffraction (XRD, Bruker D8 Advance, Bruker AXS GmbH, Karlsruhe, Germany) with Cu Kα radiation (λ = 1.5406 Å) operated at 40 kV and 40 mA. The surface elemental composition and chemical states were examined by X-ray photoelectron spectroscopy (XPS, Thermo Scientific ESCALAB 250Xi, Thermo Fisher Scientific, Waltham, MA, USA) with Al Kα radiation (hν = 1486.6 eV), and the C 1s signal at 284.8 eV was used for calibration. Optical absorption characteristics were investigated using UV–visible diffuse reflectance spectroscopy (UV–Vis DRS, Shimadzu UV-2600, Shimadzu Corp., Kyoto, Japan) with BaSO_4_ as the background, and the band gap values (Eg) were obtained from Tauc plots derived from the Kubelka–Munk function. Photoluminescence (PL) spectra were collected on a Horiba FluoroMax-4 spectrophotometer at 325 nm excitation (Horiba Scientific, Kyoto, Japan). Electrochemical impedance spectroscopy (EIS) and photocurrent response tests were carried out on a CHI660E workstation (CH Instruments, Shanghai, China) with a standard three-electrode setup: Pt wire as the counter electrode, Ag/AgCl as the reference, and the catalyst-coated indium tin oxide (ITO) glass as the working electrode.

### 2.3. Photodegradation Procedures

The photocatalytic efficiency of the synthesized materials was assessed through the degradation of organic dyes under visible light. A 300 W Xe lamp served as the irradiation source, providing an intensity of ~100 mW·cm^−2^. Typically, 30 mg of catalyst was ultrasonically dispersed in 100 mL of an aqueous dye solution (10 mg·L^−1^). Before illumination, the suspension was kept under stirring in the dark for 30 min to achieve adsorption–desorption equilibrium. During the reaction, 3 mL aliquots were taken at predetermined intervals, centrifuged to separate the catalyst, and the dye concentration was determined by monitoring the absorbance at λmax = 554 nm using a UV-Vis spectrophotometer (Shimadzu UV-2600). The degradation efficiency (η) of dyes was calculated according to the following equation [42]:

(1) $$\eta \left(\%\right)=\frac{C_{0}-C_{t}}{C_{0}}\times 100\%$$

To evaluate the durability of the photocatalysts, recycling experiments were conducted. After each cycle, the catalyst was recovered by centrifugation, thoroughly rinsed with ethanol and deionized water, and dried at 60 °C for reuse. In addition, radical scavenging experiments were performed by introducing isopropanol (IPA), benzoquinone (BQ), and ethylenediaminetetraacetic acid disodium salt (EDTA-2Na) as quenchers for •OH, •O_2_^−^, and h^+^, respectively, to identify the main reactive species involved in the photodegradation process.

## 3. Result and Discussion

### 3.1. Morphology and Structural Properties

The morphology and microstructure of the TiO_2_-based composites were systematically investigated by SEM and TEM. As shown in Figure 1b,c, the pristine TiO_2_ microspheres exhibit well-defined spherical architectures composed of numerous nanosheets, resulting in a hierarchical porous structure that is expected to provide abundant active sites for photocatalytic reactions. After the incorporation of Ag nanoparticles, the microspheres retain their spherical appearance, while the interparticle voids are uniformly decorated with Ag nanoparticles (Figure 1d,e). Upon further hybridization with RGO, the Ag-TiO_2_ microspheres are uniformly wrapped by thin RGO nanosheets (Figure 1f), which effectively suppress particle aggregation and construct a conductive framework for charge transport. Elemental mapping (Figure 1g) confirms the homogeneous distribution of C, Ti, O, and Ag, verifying the successful integration of RGO and Ag nanoparticles with TiO_2_. TEM and HRTEM analyses provide additional structural insights: Ag-TiO_2_ nanoparticles are firmly anchored onto the flexible RGO sheets, forming close interfacial contact (Figure 1h). The HRTEM image (Figure 1i) further reveals distinct lattice fringes with interplanar spacings of 0.236 nm and 0.352 nm, corresponding to the (110) plane of metallic Ag and the (101) plane of anatase TiO_2_, respectively. These observations unambiguously confirm the successful decoration of Ag nanoparticles on TiO_2_ and their intimate coupling with RGO, which are anticipated to synergistically promote charge separation and transfer during photocatalysis process.

**Figure 2:**
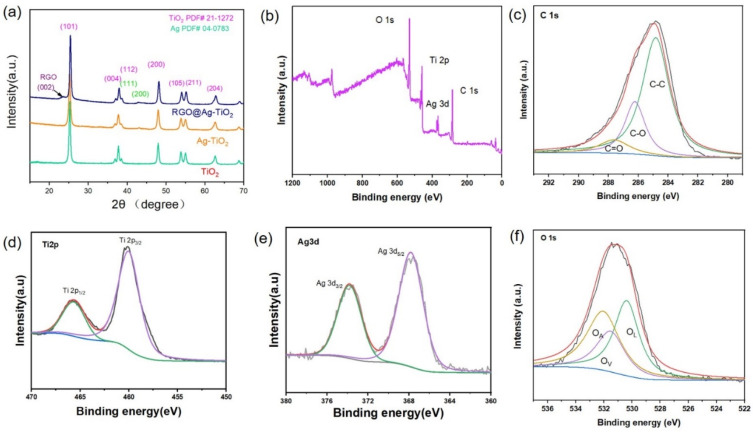
(**a**) XRD pattern of various TiO_2_-based samples; (**b**) XPS full survey spectra, (**c**) C 1s, (**d**) Ti 2p, (**e**) Ag 3d and (**f**) O 1s spectra.

The crystalline structures of the as-prepared samples were examined by XRD (Figure 2a). The characteristic peaks located at 25.3°, 37.8°, 48.0°, 53.9°, 55.1° and 62.7° can be indexed to the (101), (004), (200), (105), (211) and (204) planes of anatase TiO_2_ (JCPDS No. 21-1272), confirming that the main crystalline phase of TiO_2_ is anatase. In addition, the diffraction peaks observed at 38.1° and 44.3° correspond to the (111) and (200) planes of face-centered cubic Ag (JCPDS No. 04-0783), which indicates the successful deposition of metallic Ag nanoparticles on TiO_2_. Moreover, the broad peak around 28° corresponds to the (002) plane of reduced graphene oxide (RGO), confirming the introduction of RGO sheets into the hybrid composite. To further confirm the chemical composition and the electronic states, XPS measurements were conducted. As shown in the survey spectrum (Figure 2b), the elements C, O, Ti and Ag are clearly detected in the composite. The high-resolution spectrum of C 1s (Figure 2c) exhibits three distinct peaks at 284.5 eV, 286.2 eV and 287.8 eV, corresponding to C–C, C–O and C=O bonds, respectively, confirming the presence of RGO. The Ti 2p spectrum (Figure 2d) displays two major peaks at binding energies of around 459.0 eV and 465.0 eV, which are assigned to Ti 2p_3_/_2_ and Ti 2p_1_/_2_ of Ti^4+^ in TiO_2_. Figure 2e shows two sharp peaks at 367.2 eV and 373.3 eV, attributed to Ag 3d_5_/_2_ and Ag 3d_3_/_2_, respectively, which verify the metallic state of Ag nanoparticles in the composite. The O 1s spectrum (Figure 2f) can be deconvoluted into three peaks centered at ~530.2 eV, 531.3 eV and 532.2 eV, corresponding to lattice oxygen (O_L_), oxygen vacancies (O_V_), and surface adsorbed oxygen species (O_A_), respectively. The existence of oxygen vacancies and surface oxygen species is favorable for improving photocatalytic activity by facilitating charge separation and reactant adsorption. These XPS spectra determine that Ag-TiO_2_-RGO consists of TiO_2_, metallic and C, respectively, in well agreement with HRTEM and XRD analysis.

### 3.2. Optoelectronic Properties

**Figure 3:**
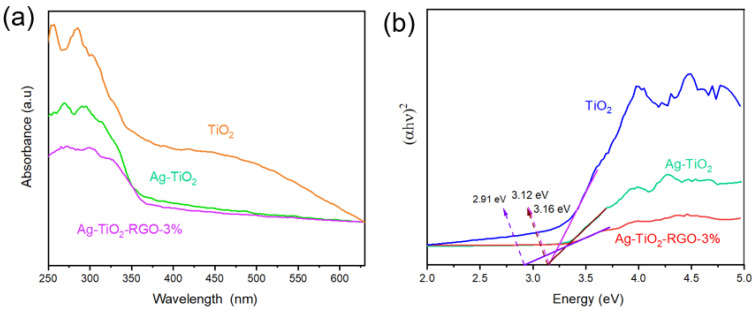
(**a**) UV–visible diffuse reflectance spectra and (**b**) band gap determination of TiO_2_, Ag-TiO_2_ and Ag-TiO_2_-RGO-3%.

The optical absorption properties of the samples were investigated by UV–visible diffuse reflectance spectroscopy (UV–Vis DRS). As shown in Figure 3a,b, pristine TiO_2_ exhibits a sharp absorption edge around 387 nm, corresponding to a band gap of ~3.16 eV, which is consistent with the intrinsic band gap of anatase TiO_2_. After deposition of Ag nanoparticles, the absorption edge of Ag-TiO_2_ remains nearly unchanged (~3.12 eV), suggesting that Ag incorporation does not significantly alter the band structure of TiO_2_ [43,44]. However, an additional broad absorption feature appears in the visible light region, which can be attributed to the surface plasmon resonance (SPR) of Ag nanoparticles, enhancing the light-harvesting ability. More importantly, for the Ag-TiO_2_-RGO composite, the absorption edge exhibits a more noticeable red shift with a reduced band gap, indicating extended absorption into the visible region. This band gap narrowing is ascribed to the strong interfacial interaction between RGO and TiO_2_, which introduces additional electronic states and facilitates electron transfer across the heterojunction [45,46]. In addition, the conductive RGO sheets serve as efficient electron acceptors and transport pathways, working synergistically with the surface plasmon resonance (SPR) effect of Ag to facilitate charge separation and enhance visible-light utilization. These findings indicate that, although UV excitation was employed for baseline photocatalytic testing, the incorporation of Ag and RGO effectively broadens the light absorption spectrum and improves the potential for visible-light-driven photocatalysis.

**Figure 4:**
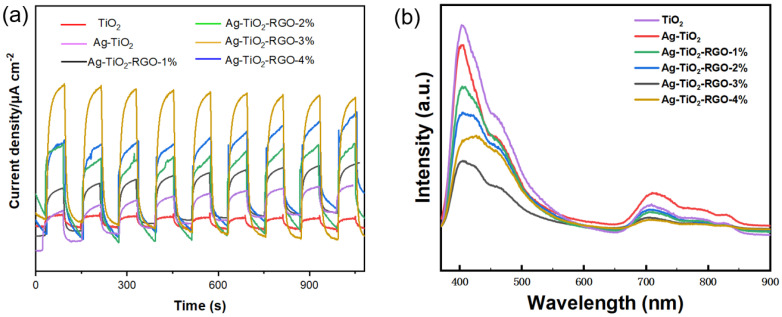
(**a**) photocurrent response and (**b**) PL spectra of TiO_2_, Ag-TiO_2_, and Ag-TiO_2_-RGO.

The transient photocurrent responses further validate the enhanced charge separation efficiency of the composites (Figure 4a). As the RGO content increases from 1 wt% to 3 wt%, the photocurrent density gradually rises and reaches a maximum at 3 wt%, which can be ascribed to an optimal balance between efficient electron-transfer pathways and sufficient light absorption by TiO_2_. At this concentration, RGO effectively suppresses electron–hole recombination while maintaining intimate contact with the semiconductor matrix [47,48]. In contrast, a further increase to 4 wt% RGO leads to a decline in photocurrent density. This reduction is likely due to the excessive coverage of TiO_2_ active sites and the shielding effect of surplus RGO layers, which hinder light harvesting and thereby suppress photocatalytic activity.

The separation and recombination behavior of photogenerated charge carriers was further investigated by photoluminescence (PL) spectroscopy (Figure 4b). Generally, a higher PL emission intensity corresponds to a higher recombination rate of photogenerated electron–hole pairs, while a lower intensity indicates more efficient charge separation. As shown in the PL spectra, pristine TiO_2_ exhibits a strong emission peak, suggesting severe recombination of photogenerated carriers. After the incorporation of Ag nanoparticles, the PL intensity of Ag-TiO_2_ decreases, implying that Ag can effectively capture electrons through the formation of a Schottky barrier, thereby suppressing recombination to some extent. The PL spectra show a decreasing trend from 1 wt% to 3 wt% RGO, with the lowest emission intensity observed at 3 wt%. This behavior suggests that moderate incorporation of RGO strongly suppresses radiative recombination of electron–hole pairs by providing efficient charge-transfer channels [49]. Nevertheless, the PL intensity increases again at 4 wt%. The excessive RGO content at high loading levels can induce restacking and aggregation, which not only weakens interfacial contact with TiO_2_ but also acts as recombination centers for photogenerated carriers. Consequently, the optimal loading of RGO is found at 3 wt%, where interfacial charge separation is maximized and recombination losses are minimized.

### 3.3. Photocatalytic Activity

**Figure 5:**
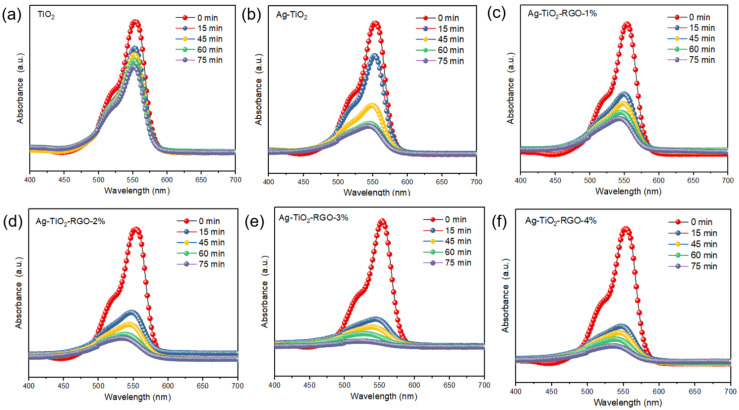
Absorbance spectra of RhB dye degradation for (**a**) TiO_2_, (**b**) Ag-TiO_2_, (**c**) Ag-TiO_2_-RGO-1%, (**d**) Ag-TiO_2_-RGO-2%, (**e**) Ag-TiO_2_-RGO-3% and (**f**) Ag-TiO_2_-RGO-4%.

The photocatalytic activities of TiO_2_, Ag-TiO_2_, and Ag-TiO_2_-RGO samples were evaluated by monitoring the degradation of RhB under visible-light irradiation (Figure 5). As shown in Figure 5a,b, pristine TiO_2_ exhibits the lowest photocatalytic activity, primarily due to its wide band gap and rapid recombination of electron–hole pairs. The introduction of Ag nanoparticles leads to a moderate improvement in performance, as Ag acts as an electron sink and facilitates charge separation through the formation of a Schottky junction at the Ag–TiO_2_ interface. Remarkably, the further incorporation of RGO results in a substantial enhancement of photocatalytic efficiency. As illustrated in Figure 5c–f, among the Ag-TiO_2_-RGO composites, the Ag-TiO_2_-RGO-3% sample demonstrates the fastest RhB degradation rate, indicating the highest photocatalytic activity. This superior performance can be attributed to synergistic effects: (i) the conductive RGO nanosheets provide a two-dimensional framework that accelerates electron transport and suppresses recombination, and (ii) Ag nanoparticles act as efficient electron traps, promoting rapid charge separation and prolonging the lifetime of charge carriers.

**Figure 6:**
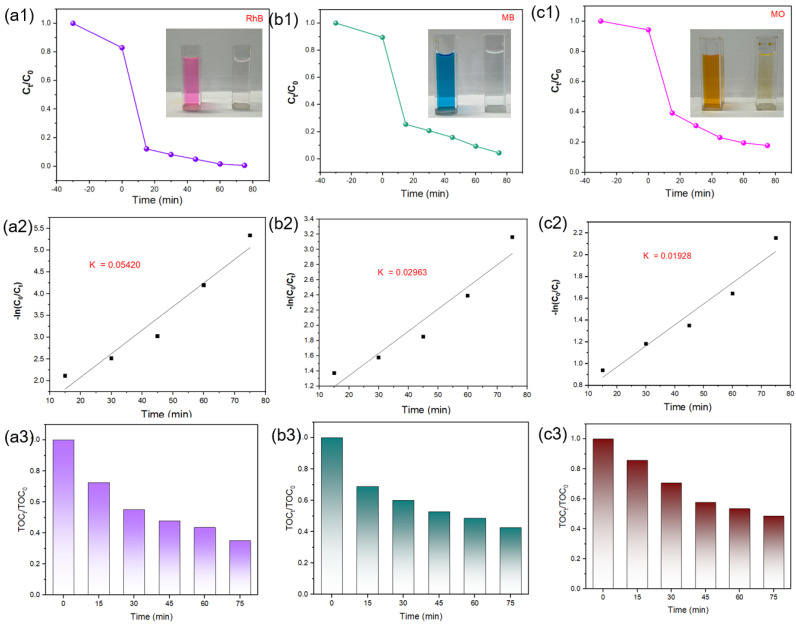
(**a1**–**c1**) Photocatalytic activity test under visible light and (**a2**–**c2**) Kinetic linear fitting results, (**a3**–**c3**) TOC analysis.

To confirm the photocatalytic capability of Ag-TiO_2_-RGO-3% to different target pollutants, the contrastive analysis of photocatalytic performance of Ag-TiO_2_-RGO-3% samples for different dyes (RhB, MB and MO) was further evaluated under visible light irradiation (Figure 6(a1–c1)). During the dark adsorption stage (−30 to 0 min), the Ag-TiO_2_-RGO-3% catalysts exhibited stronger adsorption capacity due to the large surface area and π–π interactions of RGO. Upon visible light exposure, three dyes undergo rapid decolorization, with RhB exhibiting the fastest removal rate (99.5%), followed by MB (96.3%), and MO (83.2%) showing the slowest response. Those results correspond to the digital figures (before and after degradation). This trend is further substantiated by the pseudo-first-order kinetic fitting results (Figure 6(a2–c2)), where the rate constants (k) were calculated as 0.05420 min^−1^ for RhB, 0.02963 min^−1^ for MB, and 0.01928 min^−1^ for MO, confirming the order of degradation efficiency as RhB > MB > MO. The superior photocatalytic response towards RhB can be ascribed to its strong adsorption affinity with the RGO surface through π–π stacking, which accelerates electron transfer and enhances the utilization of photogenerated reactive species. In contrast, the relatively lower reactivity of MO originates from the intrinsic stability of its azo group (–N=N–) and its weaker adsorption on the photocatalyst surface, leading to a slower degradation rate. Collectively, these findings indicate that the Ag-TiO_2_-RGO composite exhibits excellent photocatalytic activity, while its efficiency is somewhat limited for more structurally stable anionic dyes like MO. In addition to decolorization, the mineralization efficiency of Ag-TiO_2_-RGO was further examined by monitoring the total organic carbon (TOC) removal of RhB, MB, and MO solutions (Figure 6(a3–c3)). It can be observed that the TOC values decrease gradually with irradiation time for all three dyes, but the reduction rates are significantly slower compared to the rapid decline. After 75 min of irradiation, the TOC removal reached approximately 55–60% for RhB, ~50% for MB, and only ~40% for MO, indicating that although the chromophoric structures of the dyes were almost completely destroyed, a considerable amount of organic intermediates remained in the solution. This discrepancy between decolorization and mineralization highlights the fact that photocatalytic degradation is a stepwise process, in which dye molecules are first decomposed into smaller organic fragments before being further oxidized into CO_2_ and H_2_O. The relatively higher TOC removal efficiency for RhB again reflects its strong interaction with the catalyst and the more efficient electron–hole separation facilitated by Ag nanoparticles and the conductive RGO matrix. In contrast, the persistent TOC level in MO degradation suggests that the stable azo moiety leads to the accumulation of refractory intermediates, thereby hampering complete mineralization within the tested timescale. These results emphasize the necessity of combining both UV–vis and TOC analyses to comprehensively evaluate the photocatalytic performance of Ag-TiO_2_-RGO composites. To better exhibit the outstanding photocatalytic performance of Ag-TiO_2_-RGO-3%, comparative studies with relevant reports were carried out. As shown in Table 1, this work demonstrates superior photocatalytic performance in dye degradation compared with previously reported TiO_2_-based catalysts.

**Figure 7:**
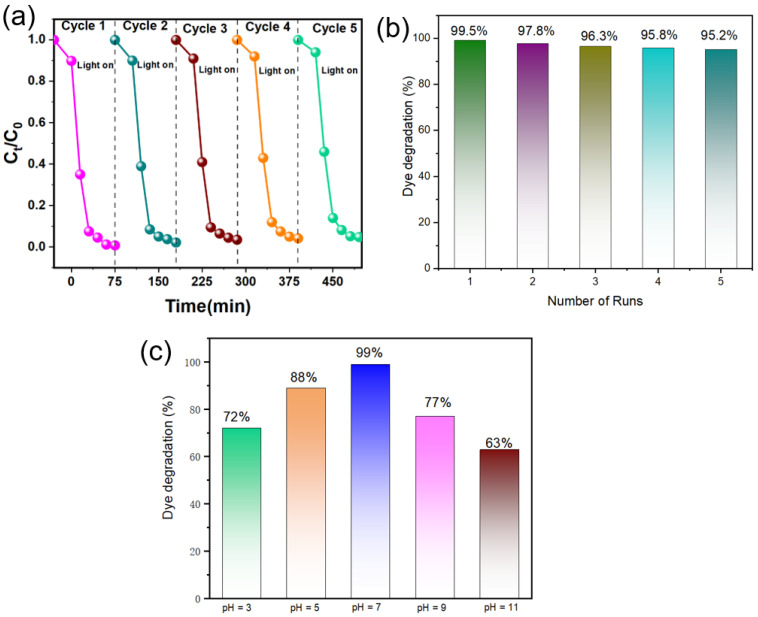
(**a**) C_t_/C_0_ curve and (**b**) histogram of cyclic stability analysis of prepared Ag-TiO_2_-RGO-3%; (**c**) Effect of pH on photocatalytic degradation of RhB for Ag-TiO_2_-RGO-3% under visible light.

**Figure 8:**
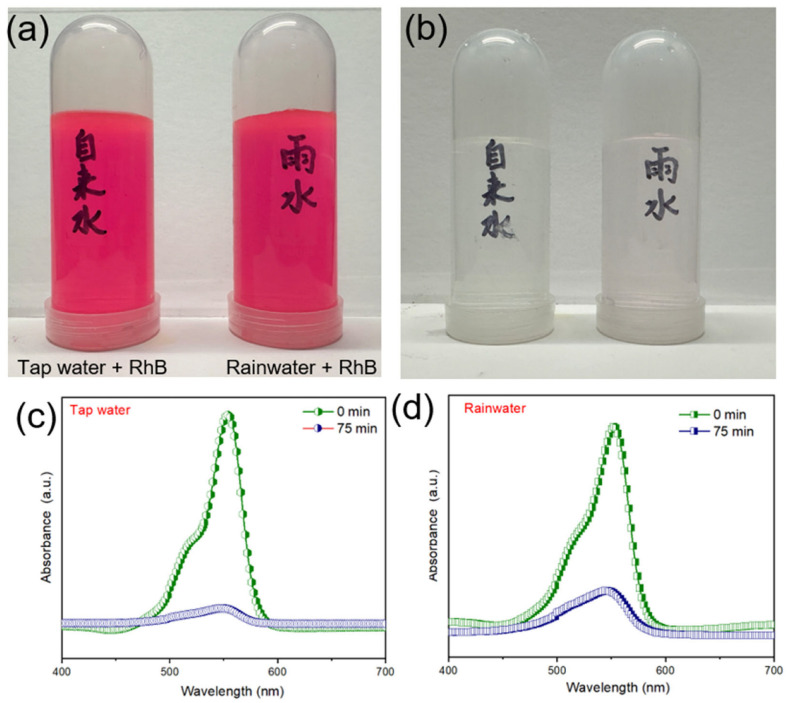
The optical images (**a**) before and (**b**) after photocatalytic decomposition of different wastewater samples containing RhB dye; Absorbance spectra of RhB dye degradation of (**c**) tap water and (**d**) rainwater.

The reusability and stability of a photocatalyst are crucial parameters for its practical application. To evaluate the cyclic stability, the photocatalytic degradation of RhB under visible light over Ag-TiO_2_-RGO-3% was tested for five consecutive cycles, with washing and drying of the catalyst between each run. As shown in Figure 7a, the C_t_/C_0_ plots over five cycles remain nearly overlapping, indicating only a slight decrease in activity. The statistical analysis in Figure 7b demonstrates that the RhB degradation efficiencies were 99.5%, 97.8%, 96.3%, 95.8%, and 95.2% for cycles 1–5, respectively. The total loss in degradation efficiency after five cycles is less than 3.5%, reflecting the excellent cyclic stability of Ag-TiO_2_-RGO-3%. This minor reduction may be attributed to partial adsorption of RhB molecules or intermediates on the catalyst surface, which could block some active sites and hinder photon-catalyst-dye interactions. Nevertheless, the strong structural stability provided by the hierarchical TiO_2_ microspheres, combined with the firm anchoring of Ag nanoparticles and RGO nanosheets, ensures long-term photocatalytic durability. The obtained cyclic performance is superior to many previously reported TiO_2_-based composites (Table 2), underlining the robustness of the designed hybrid photocatalyst. In addition to stability, the influence of solution pH on photocatalytic efficiency was also investigated, as shown in Figure 7c. The RhB degradation efficiency varies significantly with pH, being 72% (pH 3), 88% (pH 5), 99% (pH 7), 77% (pH 11), and 63% (pH 13). The best performance under neutral conditions (pH 7) can be explained by the optimal electrostatic interactions between the catalyst surface and dye molecules, as well as the favorable generation of reactive oxygen species (ROS). In acidic conditions (pH 3 and 5), protonation of dye molecules and possible dissolution/partial passivation of TiO_2_ surfaces may limit electron–hole reactions, resulting in lower activity. Under strongly alkaline conditions (pH 11 and 13), excessive hydroxyl ions may destabilize the adsorbed dye layer or induce recombination of charge carriers, again decreasing efficiency. These results highlight that while Ag-TiO_2_-RGO-3% exhibits good activity over a broad pH range, neutral pH conditions are most favorable for efficient dye degradation. To verify the performance of the catalyst under conditions closer to practical applications, we further conducted photocatalytic degradation experiments with RhB (50 mg/L) dye in different actual water sources (tap water and rainwater). As shown in Figure 8a,b, the Ag-TiO_2_-RGO-3% efficiently degraded RhB in tap water and rainwater. Obviously, the characteristic absorption peak at ~550 nm almost disappeared after 75 min of irradiation (Figure 8c). Notably, a small residual RhB signal was still observed in the rainwater system (Figure 8d), which could be attributed to the presence of natural organic matter, dissolved salts, or other competing species that partially inhibited the catalytic process. Nevertheless, the overall photocatalytic efficiency remained high, demonstrating that the performance is well maintained under more complex conditions, thereby underscoring the robustness of the catalyst and its promising applicability in real wastewater treatment.

**Figure 9:**
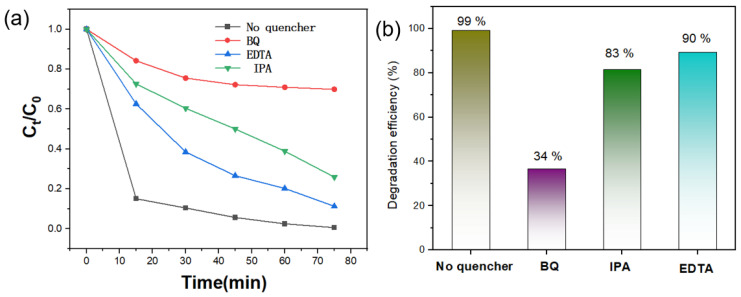
Trapping test (**a**) C/C_0_ curves (**b**) corresponding efficiency of RhB dye in the presence of various scavengers under visible light.

To further investigate the underlying photocatalytic effect of RhB degradation over the Ag-TiO_2_-RGO-3% composite, radical trapping experiments were performed to determine the primary reactive species. Different scavengers were introduced into the reaction system: benzoquinone (BQ) to quench superoxide radicals (•O_2_^−^), isopropanol (IPA) to scavenge hydroxyl radicals (•OH), and ethylenediaminetetraacetic acid (EDTA) to capture photogenerated holes (h^+^). As shown in Figure 9a,b, the addition of scavengers led to significant changes in RhB degradation efficiency compared to the control (no quencher, 99%). In the presence of BQ, the efficiency dropped drastically to 34%, indicating that •O_2_^−^ radicals are the dominant active species in the photocatalytic process. When IPA was introduced, the degradation efficiency decreased to 83%, confirming that •OH radicals also play an important but less significant role. Meanwhile, with EDTA, the efficiency was reduced to 90%, suggesting that photogenerated holes (h^+^) contribute to the reaction but are not the main active species.

These results clearly demonstrate that among the reactive species generated during the photocatalytic process, •O_2_^−^ are the primary contributors to RhB degradation, followed by •OH, while photogenerated holes (h^+^) have a relatively minor effect. The efficient generation and utilization of these radicals in Ag-TiO_2_-RGO-3% can be attributed to the synergistic effects of Ag nanoparticles, which promote surface plasmon resonance (SPR) and electron transfer, and RGO nanosheets, which enhance charge separation and suppress recombination. Therefore, these structural advantages enable the catalyst to produce abundant reactive oxygen species, thereby driving efficient RhB degradation.

### 3.4. Photodegradation Mechanism of Dyes for Ag-TiO_2_-RGO

**Figure 10:**
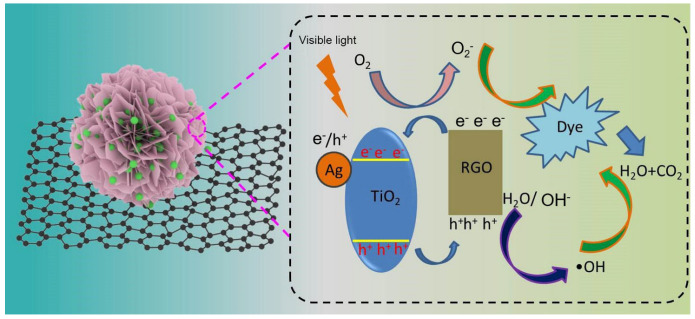
Mechanism of photocatalytic degradation of Ag-TiO_2_-RGO.

The photocatalytic degradation mechanism of RhB over Ag-TiO_2_-RGO is illustrated in Figure 10. Upon visible light irradiation, TiO_2_ nanoparticles are excited to generate electron–hole pairs (e^−^/h^+^). However, in pristine TiO_2_, these charge carriers tend to recombine rapidly, which significantly limits photocatalytic efficiency. In the ternary Ag-TiO_2_-RGO system, both Ag nanoparticles and RGO nanosheets act synergistically to promote charge separation and enhance the redox reactions. First, Ag nanoparticles deposited on the TiO_2_ surface serve as electron traps due to their lower Fermi level compared with TiO_2_, enabling photogenerated electrons to transfer from the conduction band (CB) of TiO_2_ to Ag. This suppresses e^−^/h^+^ recombination while also extending light absorption through the localized surface plasmon resonance effect of Ag. Simultaneously, it has been established that RGO possesses a stronger capacity to capture photons due to its unique 2D π-conjugation structure [62]. This structural feature enables RGO to act as an excellent electron acceptor and transporter, effectively facilitating the migration of photo-generated charge carriers. In the Ag-TiO_2_-RGO system, RGO can readily transfer the photo-excited electrons from the conduction band (CB) of TiO_2_, owing to its relatively smaller work function compared with TiO_2_. As a consequence, the recombination of electron–hole pairs is significantly suppressed, and the separation efficiency of charge carriers is remarkably improved.

The transferred electrons on Ag and RGO subsequently react with dissolved oxygen (O_2_) to produce •O_2_^−^, while photogenerated holes (h^+^) in the valence band (VB) of TiO_2_ oxidize H_2_O or surface hydroxyl groups to generate •OH. Both •O_2_^−^ and •OH are highly oxidative species capable of attacking RhB molecules, breaking down their conjugated structures and mineralizing them into CO_2_ and H_2_O [63]. Radical trapping experiments further confirmed that •OH plays a dominant role, while •O_2_^−^ and photogenerated holes (h^+^) contribute to a lesser extent. The possible reactions in the present mechanism for RhB degradation are summarized as follows [64]:

TiO_2_ + hν → TiO_2_ (e^−^ + h^+^) (2)

e^−^ (TiO_2_ CB) → e^−^ (Ag/RGO) (3)

O_2_ + e^−^ → •O_2_^−^ (4)

h^+^ + H_2_O → •OH + H^+^ (5)

h^+^ + OH^−^ → •OH (6)

RhB + •O_2_^−^/•OH → CO_2_ + H_2_O (7)

Therefore, the superior photocatalytic performance of Ag-TiO_2_-RGO can be attributed to the combined effects of Ag nanoparticles enhancing light absorption and electron trapping, RGO accelerating electron transport, and the efficient generation of •O_2_^−^ and •OH radicals that drive the oxidative decomposition of RhB into harmless end products.

## 4. Conclusions

In this study, Ag-TiO_2_-RGO-3% composites were successfully synthesized and applied for the photocatalytic degradation of dyes. Structural and optical characterizations confirmed the effective incorporation of Ag nanoparticles and RGO, which synergistically enhanced charge separation, light absorption, and reactive oxygen species (ROS) generation. Photocatalytic evaluation revealed that Ag-TiO_2_-RGO-3% achieved the highest activity, with a degradation efficiency of 99.5% and a rate constant of 0.05420 min^−1^. The material also demonstrated excellent cyclic stability over five consecutive runs and maintained robust performance under varying pH conditions. Radical scavenging experiments identified •O_2_^−^ as the dominant reactive species, with additional contributions from •OH and h^+^. Based on these findings, a photocatalytic mechanism was proposed, emphasizing the crucial roles of Ag and RGO in facilitating charge transfer and enhancing ROS generation. Overall, this work demonstrates that Ag-TiO_2_-RGO is an efficient and durable photocatalyst with strong potential for practical wastewater treatment and environmental remediation. Looking ahead, the use of biomass-derived graphene oxide could further improve the sustainability of the system, thereby reinforcing its environmental relevance and scalability for real-world applications.

## Figures and Tables

**Table 1:** Comparative analysis on photocatalytic degradation of dye of TiO_2_-based Catalysis.

Catalyst	Light Source	Dye	Irradiation Time (min)	Degradation (%)	Ref.
TiO_2_	UV	MB	40	86	[50]
TiO_2_	UV	RhB	120	96	[51]
TiO_2_	Visible light	MB	120	93	[52]
RGO/TiO_2_	UV	MO	180	94	[53]
Ag/TiO_2_	Visible light	RhB	150	79	[54]
Ag-Ag_2_O/TiO_2_@PP	UV	MB	120	83	[55]
Ag_2_O/ZnO–TiO_2_	Visible light	RhB	90	30	[56]
Ag-TiO_2_-RGO-3%	Visible light	RhB	75	99	This work

**Table 2:** Comparison of photocatalytic cycle performance of TiO_2_-based catalytic with reported literature.

Catalytic	Cycle Times	Dye	Dye Degradation (%)	Ref.
TiO_2_-Ex-rGO	3	MB	95	[57]
TiO_2_	5	MO	67	[58]
TiO_2_-SWCNT	4	RhB	93	[59]
Mn-B-TiO_2_	3	RhB	95	[60]
TiO_2_/g-C_3_N_4_	4	RhB	88	[61]
Ag-TiO_2_-RGO-3%	5	RhB	96	This work

## Data Availability

Data are contained within the article.
